# Vitamin D deficiency and atopic dermatitis severity in a Bangladeshi population living in East London: A cross‐sectional study

**DOI:** 10.1002/ski2.358

**Published:** 2024-03-12

**Authors:** Rebecca L. McCarthy, Soha S. Tawfik, Ioannis Theocharopoulos, Ravinder Atkar, Bryan McDonald, Sasha Dhoat, Aaron Hughes, Bjorn R. Thomas, Edel A. O’Toole

**Affiliations:** ^1^ Dermatology Department The Royal London Hospital Barts Health NHS Trust London UK; ^2^ Blizard Institute The Faculty of Medicine and Dentistry Queen Mary University of London London UK; ^3^ Department of Dermatology Venereology and Andrology Faculty of Medicine Alexandria University Alexandria Egypt

## Abstract

**Background:**

Atopic eczema is a common, chronic, inflammatory skin condition with considerable heterogeneity. South Asian people living in the UK frequently have low serum vitamin D3 (25(OH)D_3_), and those with atopic disease can present with severe eczema. The association between vitamin D deficiency and eczema severity, and the role of vitamin D supplementation in atopic eczema is inconsistent, and under‐researched in people with Asian ancestry.

**Objectives:**

This cross‐sectional study investigates the association between serum 25(OH)D_3_ and eczema severity in a cohort of South Asian children and young adults living in London.

**Methods:**

Eligible participants were Bangladeshi children and young adults aged 0–30 years with eczema, living in London and participating in the Tower Hamlets Eczema Assessment study. Data was collected via parent/patient self‐reporting, clinical history and examination, and hospital databases. 25(OH)D_3_ levels were documented retrospectively, if available, from hospital databases. Eczema severity was classified by Eczema Area and Severity Index (EASI) score less than or greater than 10 (clear‐mild vs. moderate‐severe). Multivariate logistic regression was used to adjust for confounding factors.

**Results:**

681 participants were included in analyses. 25(OH)D_3_ results were available for 49.6% (338/681), 84.3% of which had deficient or insufficient lowest 25(OH)D_3_. Lowest 25(OH)D_3_ was inversely correlated with EASI score (Spearman's rank *R* ^2^ = −0.24, *p* < 0.001). 26.1% (178/681) had EASI >10 and a lower median lowest and nearest 25(OH)D_3_. After adjustment for confounding EASI > 10 was significantly associated with a lowest 25(OH)D_3_ < 25 (OR 3.21, 95%CI 1.35, 8.60), use of mild‐moderate potency topical steroid on the face and neck (OR 3.11, 95%CI 1.86, 5.31), calcineurin inhibitor on the face and neck (OR 2.79, 95% CI 1.26, 6.10) and potent – very potent topical steroid on the face and neck (OR2.23, 95%CI 1.02, 4.77) and body (OR 2.11, 95%CI 1.18, 3.87).

**Discussion:**

Vitamin D plays a role in modulation of proteins required for skin barrier function and regulation of the innate immune system, suggesting 25(OH)D_3_ deficiency contributes to skin inflammation. This study demonstrates a relationship between 25(OH)D_3_ deficiency and worse eczema severity in a cohort of South Asian children and young adults.

## Graphical abstract

This cross‐sectional study investigates the association between serum vitamin D levels and eczema severity in a cohort of South Asian children and young adults living in London. The results support that vitamin D deficiency is associated with worse eczema severity, after adjustment for confounding factors.

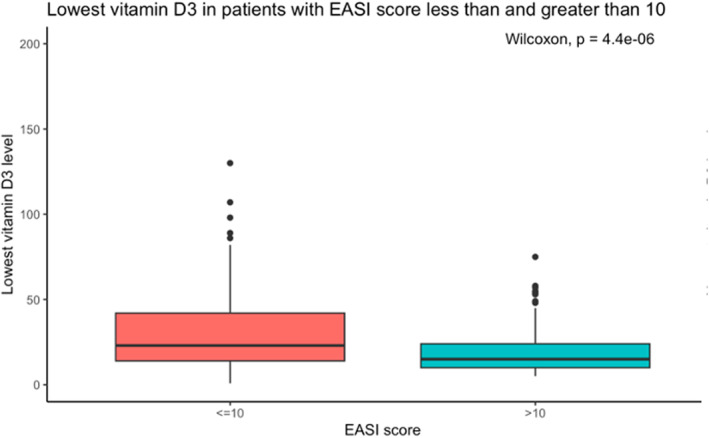

**What is already known about this topic?**

- South Asian people living in the UK have lower serum vitamin D3 levels compared to white people living in the UK and compared to South Asian people living in South Asia.
- A growing body of research is investigating the relationship between vitamin D3 and atopic diseases. There is evidence of lower vitamin D3 levels in atopic eczema, however, the association between vitamin D and eczema severity is conflicted.

**What does this study add?**

- This study assesses the relationship between Eczema Area and Severity Index (EASI) score and serum vitamin D3 in a large cohort of Bangladeshi patients, who are under‐studied in existing studies of the role of vitamin D supplementation in eczema.
- The results demonstrate a relationship between vitamin D3 deficiency and worse eczema severity in South Asian children and young adults.

## 1 BACKGROUND

Human skin pigmentation plays a pivotal role in mediating penetration of ultraviolet (UV) radiation into the deep layers of skin and the cutaneous circulation.^1^ Depigmented skin has evolved in high latitude regions with low average UV light, such as the UK, facilitating cutaneous vitamin D production.^1^, ^2^ It is well documented that South Asian people living in the UK have lower serum 25‐hydroxy vitamin D3 (25(OH)D_3_) compared to white people living in the UK and compared to South Asian people living in South Asia.^3^, ^4^, ^5^, ^6^

Vitamin D plays a key role in the innate and adaptive immune systems. A growing body of research is investigating the relationship between vitamin D and atopic diseases. There is evidence of lower vitamin D levels in atopic eczema,^5^ however, the association between vitamin D and eczema severity is conflicted. In addition, there is inconclusive evidence on the benefit of vitamin D supplementation in atopic eczema.^7^, ^8^

The Tower Hamlets borough in London has a large Bangladeshi population, with 32% of the population identifying as Bangladeshi in 2018.^9^ Anecdotally, atopic eczema in this population is severe, often requiring systemic treatment, and it is frequently complicated by eczema herpeticum. Filaggrin (*FLG*) is the major atopic eczema risk gene in the Bangladeshi population, and there is increased variation in *FLG* mutations.^10^ We hypothesise that there is a relationship between eczema severity and vitamin D deficiency in this population.

The primary aim of this study was to determine the relationship between serum 25(OH)D_3_ and eczema severity in a population of Bangladeshi children and young adults living in East London.

## 2 MATERIALS AND METHODS

### 2.1 Regulatory approval

Ethical approval was granted through the Health Research Authority after review by the Hampstead Regional Ethics Committee (REC 18/LO/0017; ReDA reference: 011978).

### 2.2 Study and subjects

This cross‐sectional study forms part of the Tower Hamlets Eczema Assessment (THEA) study. All recruitment took place through paediatric and adult dermatology clinics, and paediatric allergy clinics, at The Royal London Hospital, London, UK. Bangladeshi children and young adults aged 0–30 years, living in East London, and with a diagnosis of atopic eczema confirmed by two dermatologists, were eligible for the study. Participants were considered Bangladeshi if both parents were from Bangladesh or if both sets of grandparents were from Bangladesh. Patients with known congenital recessive or x‐linked ichthyoses, mixed ethnicities or equivocal diagnosis of atopic eczema were excluded. Participation included a once only study visit where data collection including questionnaires and physical examination were performed, and other medical history data was collected retrospectively from hospital databases.

Participants were recruited between May 2018 and December 2021. Data were collected by a Bangladeshi research assistant or research nurse, and a dermatology consultant or registrar. Written, informed consent or assent was obtained from the patient and/or their parent(s) prior to recruitment. For participants and/or parents who could not read or write in English, informed consent and data collection was performed in either spoken Bengali or Sylheti.

### 2.3 Outcome

Personal report and hospital records of admission with bacterial infection and eczema herpeticum were recorded. Data was collected on current or prior use of immunosuppression/biologic therapy or prophylactic antibiotics/antiviral medications. 25(OH)D_3_ levels were recorded retrospectively where available in hospital databases. Lowest ever recorded 25(OH)D_3_ was recorded as ‘lowest 25(OH)D_3_’, and serum 25(OH)D_3_ closest to the patient recruitment date was recorded as ‘nearest 25(OH)D_3_’. If only one 25(OH)D_3_ level was available, it was used for both nearest and lowest levels. 25(OH)D_3_ levels were classified as deficient (<25 nmol/L), insufficient (25–50 nmol/L) and sufficient (>50 nmol/L).^11^

Eczema severity was classified by Eczema Area and Severity Index (EASI) score, which is a validated tool for clinical studies investigating atopic eczema.^12^, ^13^ EASI score was calculated during the hospital visit. The primary outcome was the probability of having an EASI score >10 with a history of vitamin D deficiency. EASI score was dichotomized as EASI ≤ 10 (clear‐mild) and EASI > 10 (moderate‐severe) based upon previous data generated by the cohort, as described by Thomas *et al.* ^14^ EASI scores are often dichotomised into EASI ≤ 7 and EASI >7.^12^, ^15^ Analysis of the relationship between serum vitamin D3 and EASI scores ≤ 7 and > 7 are available in supplementary information (Tables S1, S2, S4 & S5, Figure S3).

### 2.4 Saliva collection for genomic DNA and targeted FLG sequencing

A sample of saliva was used to collect DNA for the project (GeneFixTM DNA Saliva Collectors from Isohelix, Harrietsham, UK).

### 2.5 Statistical analysis

Descriptive statistics are presented as counts and percentages for categorical variables and mean and standard deviation for continuous variables. Chi‐squared, Fisher's exact or Wilcoxon rank‐sum tests were used for bivariate analysis.

A binomial generalized linear model was fitted with EASI score (EASI ≤ 10 and EASI > 10) as the dependent variable. Variables were selected for inclusion based upon demographic features (age, sex, BMI, socio‐economic class^16^) and plausible predictive variables: lowest serum 25(OH)D_3_,^17^ personal history of atopic disease,^18^ current topical anti‐inflammatory treatment on head and body, current and previous use of immunosuppressive therapy, and *FLG* loss of function mutations (*FLG* LOFM).^19^

All statistical analyses were performed using R (version 4.1.2). *p*‐values ≤ 0.05 were considered statistically significant. Full details of collection of saliva samples, DNA extraction and analysis and handling of missing data is available in supplementary information (Appendix 1).

## 3 RESULTS

### 3.1 Demographic data

682 participants were recruited to the THEA study between May 2018 and December 2021. One participant is excluded from analyses due to missing EASI score. Baseline demographics are summarised in Table 1. Mean age at recruitment was 9.5 years (interquartile range (IQR) 4.8, 14.5) and 57% (391/681) of participants were male. All participants were of Bangladeshi descent and 97% (660/681) were born in the UK.

**TABLE 1:** Baseline characteristics of whole cohort and by clear‐mild versus moderate‐severe eczema.

Characteristic	N	Overall *N* = 681^a^	Eczema severity		*p*‐value^b^	*q*‐value^c^
			Clear – mild^d^ *N* = 503^a^	Moderate ‐severe^d^ *N* = 178^a^		
Age	681	9.5 (4.8, 14.5)	8.9 (4.4, 14.1)	11.4 (6.3, 15.9)	**<0.001**	**0.010**
Male sex	681	391 (57%)	277 (55%)	114 (64%)	**0.037**	0.86
BMI	673	17.9 (15.6, 21.9)	17.8 (15.6, 21.7)	18.3 (15.6, 22.9)	0.40	>0.99
Missing		8	6	2		
Country of birth	681				0.66	>0.99
UK		660 (97%)	489 (97%)	171 (96%)		
Bangladesh		3 (0.4%)	2 (0.4%)	1 (0.6%)		
Other		18 (2.6%)	12 (2.4%)	6 (3.4%)		
ESEC	679				**0.046**	>0.99
Intermediate		189 (28%)	128 (26%)	61 (34%)		
Salariat		146 (22%)	116 (23%)	30 (17%)		
Working		344 (51%)	257 (51%)	87 (49%)		
Missing		2	2	0		
EASI score	681	4.3 (1.5, 10.4)	2.8 (1.2, 5.4)	17.7 (13.1, 25.1)	**<0.001**	**<0.001**
POEM score	681	13.0 (8.0, 19.0)	11.0 (6.0, 17.0)	18.5 (14.0, 22.8)	**<0.001**	**<0.001**
DLQI	110	7.0 (3.0, 14.0)	5.0 (3.0, 8.0)	15.0 (8.0, 18.0)	**<0.001**	**<0.001**
Missing		571	430	141		
CDLQI	430	9.0 (5.0, 14.0)	7.0 (4.0, 13.0)	13.0 (9.0, 17.0)	**<0.001**	**<0.001**
Missing		251	187	64		
Lowest 25(OH)D_3_	338	21.0 (13.0, 37.8)	23.0 (14.0, 42.8)	15.0 (10.0, 24.0)	**<0.001**	**<0.001**
Missing		343	273	70		
Nearest 25(OH)D_3_	338	31.0 (19.0, 51.0)	33.0 (21.0, 54.0)	27.0 (16.8, 46.2)	**0.016**	0.36
Missing		343	273	70		
Admission to hospital with eczema flare	679	48 (7.1%)	23 (4.6%)	25 (14%)	**<0.001**	**<0.001**
Missing		2	2	0		
Admission to hospital with eczema herpeticum	680	99 (15%)	55 (11%)	44 (25%)	**<0.001**	**<0.001**
Missing		1	1	0		
Admission to hospital with bacterial infection	680	46 (6.8%)	21 (4.2%)	25 (14%)	**<0.001**	**<0.001**
Missing		1	1	0		
Current immunosuppressive therapy	681	34 (5.0%)	13 (2.6%)	21 (12%)	**<0.001**	**<0.001**
Previous immunosuppressive therapy	681				**<0.001**	**<0.001**
Never		605 (89%)	467 (93%)	138 (78%)		
Previous or current		76 (11%)	36 (7.2%)	40 (22%)		
Current prophylactic antibiotics or antivirals	681	20 (2.9%)	10 (2.0%)	10 (5.6%)	**0.014**	0.31
Topical anti‐inflammatory use on head/neck	681				**<0.001**	**<0.001**
No anti‐inflammatory treatment		269 (40%)	235 (47%)	34 (19%)		
Mild ‐ moderate steroid		305 (45%)	197 (39%)	108 (61%)		
Potent ‐ very potent steroid		51 (7.5%)	35 (7.0%)	16 (9.0%)		
Calcineurin inhibitor		49 (7.2%)	31 (6.2%)	18 (10%)		
Other		7 (1.0%)	5 (1.0%)	2 (1.1%)		
Topical anti‐inflammatory use on body	681				**<0.001**	**<0.001**
No anti‐inflammatory treatment		179 (26%)	158 (31%)	21 (12%)		
Mild ‐ moderate steroid		99 (15%)	87 (17%)	12 (6.7%)		
Potent ‐ very potent steroid		390 (57%)	249 (50%)	141 (79%)		
Calcineurin inhibitor		6 (0.9%)	4 (0.8%)	2 (1.1%)		
Other		7 (1.0%)	5 (1.0%)	2 (1.1%)		
Physician diagnosed asthma	681	171 (25%)	114 (23%)	57 (32%)	**0.013**	0.31
Physician diagnosed allergic rhinitis	681	214 (31%)	156 (31%)	58 (33%)	0.70	>0.99
Physician diagnosed food allergy	681	276 (41%)	200 (40%)	76 (43%)	0.49	>0.99
FLG LOFM	681				0.36	>0.99
None		387 (57%)	293 (58%)	94 (53%)		
At least one		227 (33%)	160 (32%)	67 (38%)		
Not tested		67 (9.8%)	50 (9.9%)	17 (9.6%)		

*Note*: Values in bold considered statistically significant.

Abbreviations: CDLQI, Children's Dermatology Life Quality Index; DLQI, Dermatology Life Quality Index; EASI, Eczema Area and Severity Index; ESEC, European Socioeconomic Class; FLG LOFM, *FLG* loss of function mutation; POEM, Patient Orientated Eczema Measure.

^a^ Median (IQR); *n* (%).

^b^ Wilcoxon rank sum test; Pearson's Chi‐squared test; Fisher's exact test.

^c^ Bonferroni correction for multiple testing.

^d^ Clear‐mild defined as EASI score ≤10, moderate‐severe eczema defined as EASI score >10.

Of all participants, 6.8% (46/680) had been admitted to hospital for bacterial infections, 15% (99/680) with eczema herpeticum and 7.1% (48/679) with eczema flare. 5.0% (34/682) were on immunosuppressive or biologic therapy at the time of data collection, 11% (76/681) had previously been on immunosuppression/biologic therapy, and 2.9% (20/681) were currently taking prophylactic antibiotics or antivirals for eczema.

**FIGURE 1:**
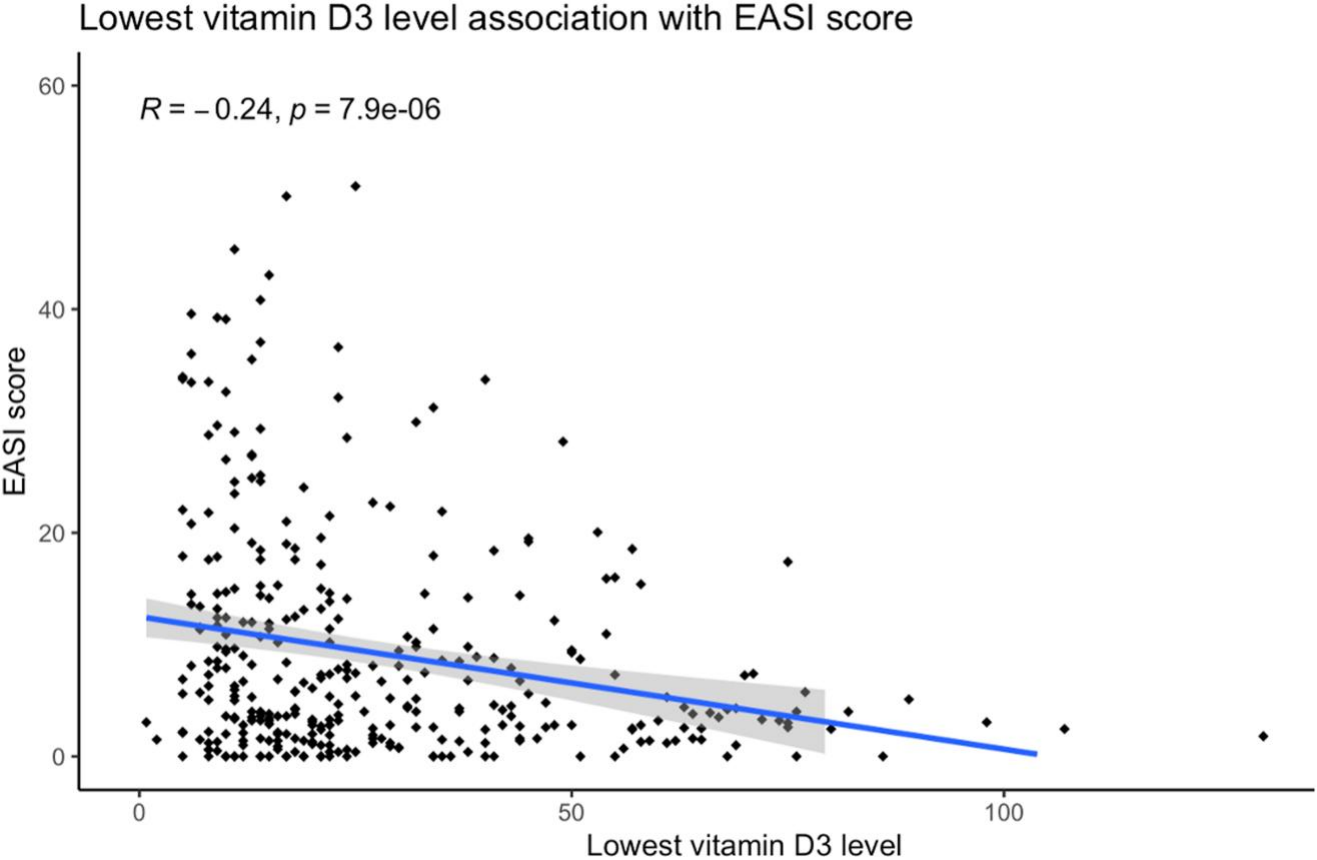
Correlation between Eczema Area and Severity Index (EASI) score and lowest vitamin D3 level. Lowest 25(OH)D_3_ is inversely related to EASI score Spearman's rank *R* ^2^ = −0.22, *p* < 0.001.

Serum 25(OH)D_3_ results were available for 49.6% (338/681). Median lowest 25(OH)D_3_ was 21.0 (IQR 13.0, 37.8) and median nearest 25(OH)D_3_ was 31.0 (IQR 19.0,51.0). Of those with 25(OH)D_3_ testing, 84.3% (285/338) had a lowest 25(OH)D_3_ result less than or equal to 50% and 73.1% (147/338) had a nearest 25(OH)D_3_ less than or equal to 50. There was no significant difference between serum 25(OH)D_3_ and month or season of testing. Median EASI score was 4.3 (1.5, 10.4). Median EASI score was higher in those deficient and insufficient in 25(OH)D_3_ than in those with sufficient 25(OH)D_3_ and in the group with no available serum 25(OH)D_3_ (Table 2). Distribution of eczema severity and 25(OH)D_3_ are displayed in Figure S1. Both lowest and nearest 25(OH)D_3_ were inversely correlated with EASI score (lowest 25(OH)D_3_ Spearman's rank *R* ^2^ = −0.24, *p* < 0.001, Figure 1; nearest 25(OH)D_3_ Spearman's rank *R* ^2^ = −0.11, *p* = 0.035, Figure S2).

**TABLE 2:** Baseline characteristics of whole cohort and categorised by lowest vitamin D3.

Characteristic	N	Overall *N* = 681^a^	Lowest serum 25(OH)D_3_				*p*‐value^b^
			≥50 *N* = 53^a^	25 – 50 *N* = 81^a^	<25 *N* = 204^a^	Not tested *N* = 343^a^	
Age	681	9.5 (4.8, 14.5)	6.0 (4.2, 8.7)	10.1 (6.8, 14.4)	13.0 (9.3, 16.5)	7.2 (3.0, 13.2)	**<0.001**
Male sex	681	391 (57%)	30 (57%)	45 (56%)	122 (60%)	194 (57%)	0.87
BMI	673	17.9 (15.6, 21.9)	16.3 (15.2, 17.9)	19.0 (16.0, 21.7)	20.0 (16.5, 23.7)	17.4 (15.3, 21.9)	**<0.001**
Missing		8	0	1	3	4	
ESEC	679						0.20
Working		344 (51%)	24 (45%)	40 (50%)	107 (53%)	173 (50%)	
Intermediate		189 (28%)	17 (32%)	19 (24%)	65 (32%)	88 (26%)	
Salariat		146 (22%)	12 (23%)	21 (26%)	31 (15%)	82 (24%)	
Missing		2	0	1	1	0	
History of atopic disease	681	509 (75%)	42 (79%)	62 (77%)	179 (88%)	226 (66%)	**<0.001**
EASI score	681	4.3 (1.5, 10.4)	3.2 (1.8, 5.8)	4.6 (1.6, 9.8)	7.0 (2.2, 15.0)	3.7 (1.2, 9.0)	**<0.001**
EASI score category	681						**<0.001**
≤ 7		421 (62%)	40 (75%)	50 (62%)	104 (51%)	227 (66%)	
>7		260 (38%)	13 (25%)	31 (38%)	100 (49%)	116 (34%)	
EASI score category	681						**<0.001**
≤ 10		503 (74%)	46 (87%)	62 (77%)	122 (60%)	273 (80%)	
>10		178 (26%)	7 (13%)	19 (23%)	82 (40%)	70 (20%)	
Admission with eczema herpeticum	680						**0.016**
Never		581 (85%)	43 (81%)	69 (85%)	163 (80%)	306 (89%)	
At least once		99 (15%)	10 (19%)	12 (15%)	41 (20%)	36 (11%)	
Missing		1	0	0	0	1	
Admission with bacterial infection	680						**0.008**
Never		634 (93%)	50 (94%)	76 (94%)	180 (88%)	328 (96%)	
At least once		46 (6.8%)	3 (5.7%)	5 (6.2%)	24 (12%)	14 (4.1%)	
Missing		1	0	0	0	1	
Admission with eczema flare	679						0.22
Never		631 (93%)	51 (96%)	71 (88%)	189 (93%)	320 (94%)	
At least once		48 (7.1%)	2 (3.8%)	10 (12%)	15 (7.4%)	21 (6.2%)	
Missing		2	0	0	0	2	
*FLG* LOFM	681						0.080
None		347 (51%)	26 (49%)	35 (43%)	113 (55%)	173 (50%)	
At least one		204 (30%)	16 (30%)	29 (36%)	66 (32%)	93 (27%)	
Not tested		130 (19%)	11 (21%)	17 (21%)	25 (12%)	77 (22%)	

*Note*: Values in bold considered statistically significant.

Abbreviations: EASI, Eczema Area and Severity Index; ESEC, European Socioeconomic Class; FLG LOFM, FLG loss of function mutation; POEM, Patient Orientated Eczema Measure.

^a^ Median (IQR); *n* (%).

^b^ Kruskal‐Wallis rank sum test; Pearson's Chi‐squared test; Fisher's exact test.

### 3.2 Vitamin D3 and eczema severity

**FIGURE 2:**
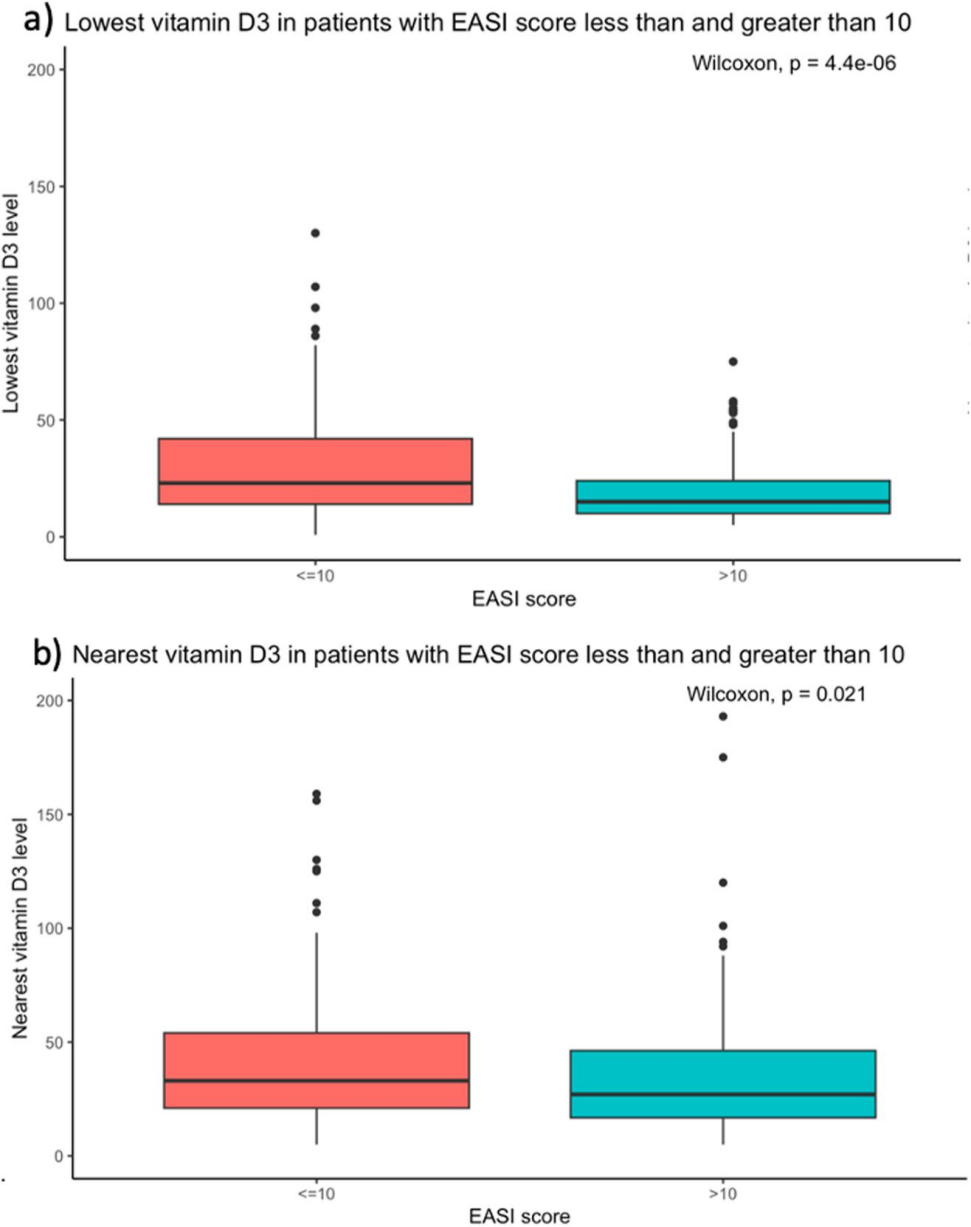
Box plots of (a) lowest and (b) nearest vitamin D3 levels in participants with Eczema Area and Severity Index (EASI) ≤10 and EASI >10.

When dichotomized into clear‐mild versus moderate‐severe eczema, 26.1% (178/681) had moderate‐severe eczema (Table 1). Those with moderate‐severe eczema had a lower median lowest (15.0 vs. 23.0, *p* < 0.001) and nearest (27.0 vs. 33.0, *p* = 0.016) serum 25(OH)D_3_ (Figure 2). After adjustment for multiple comparisons, EASI >10 was seen in older patients (*p* = 0.010), patients with lower lowest‐ever 25(OH)D_3_ (*p* < 0.001), increased history of admission to hospital due to bacterial skin infection (*p* < 0.001), eczema herpeticum (*p* < 0.001) and eczema flare (*p* < 0.001) and associated with current (*p* < 0.001) and previous (*p* < 0.001) use of immunosuppressive or biologic therapy, and current use of topical anti‐inflammatory therapy on the head and neck (*p* < 0.001) and body (*p* < 0.001) (Table 1). The presence of at least one *FLG* LOFM was associated with higher median EASI score (4.0 vs. 6.0, *p* = 0.008). There was no difference between *FLG* LOFM status and serum 25(OH)D_3_ (lowest 25(OH)D_3_ 19.5 versus 22.0, *p* = 0.15; nearest 25(OH)D_3_ 31.0 versus 29.0, *p* = 0.34) (Table S3).

After adjustment for confounding factors using logistic regression, variables associated with EASI > 10 included a vitamin D3 deficiency (lowest 25(OH)D_3_ < 25, OR 3.26, 95%CI 1.35, 8.60; *p* = 0.012), mild‐moderate potency topical steroid use on the face and neck (OR 3.11, 95%CI 1.86, 5.31; *p* < 0.001), topical calcineurin inhibitor use on face and neck (OR 2.79, 95% CI 1.26, 6.10, *p* = 0.010) and potent – very potent topical steroid use on the head and neck (OR2.23, 95%CI 1.02, 4.77, *p* = 0.041) and body (OR 2.09, 95%CI 1.17, 3.83; *p* = 0.015) (Table 3).

**TABLE 3:** Odds ratio of EASI >10 using lowest vitamin D3.

Characteristic	OR	95% CI	*p*‐value
Lowest 25(OH)D_3_ (nmol/L)			
≥ 50 (sufficient)		Reference	
25 – 50 (insufficient)	1.70	0.64, 4.91	0.3
< 25 (deficient)	3.21	1.35, 8.60	**0.012**
Not tested	1.86	0.81, 4.86	0.2
Age	1.04	0.99, 1.08	0.10
Male sex	1.44	0.97, 2.14	0.072
BMI	0.98	0.93, 1.03	0.4
History of atopic disease	0.86	0.53, 1.40	0.5
Current immunosuppressive therapy	1.66	0.73, 3.86	0.2
Previous immunosuppressive therapy	1.80	0.98, 3.30	0.056
Topical anti‐inflammatory treatment on head/neck			
No anti‐inflammatory treatment		Reference	
Mild ‐ moderate steroid	3.11	1.86, 5.31	**<0.001**
Potent ‐ very potent steroid	2.23	1.02, 4.77	**0.041**
Calcineurin inhibitor	2.79	1.26, 6.10	**0.010**
Other	2.13	0.23, 13.1	0.4
Topical anti‐inflammatory treatment on body			
No anti‐inflammatory treatment		Reference	
Mild ‐ moderate steroid	0.69	0.29, 1.56	0.4
Potent ‐ very potent steroid	2.09	1.17, 3.83	**0.015**
Calcineurin inhibitor	1.09	0.13, 6.91	>0.9
Other	1.72	0.22, 9.70	0.6
ESEC			
Working		Reference	
Intermediate	1.41	0.91, 2.20	0.12
Salariat	0.92	0.54, 1.54	0.8
Any FLG LOFM			
None		Reference	
At least one	1.32	0.85, 2.03	0.2
Not tested	1.07	0.62, 1.82	0.8
No. Obs.	671		
Log‐likelihood	−327		
AIC	698		
BIC	798		

*Note*: Values in bold considered statistically significant.

Abbreviations: AIC, Akaike information criterion; BIC, Bayesian information criterion; CI, confidence interval; EASI, Eczema Area and Severity Index; ESEC, European Socioeconomic Class; FLG LOFM, FLG loss of function mutation; OR, odds ratio.

## 4 DISCUSSION

Our data has demonstrated a relationship between lowest 25(OH)D_3_ and worse eczema severity in a Bangladeshi population living in East London. After adjusting for confounding factors including age, sex, BMI, socio‐economic class, history of atopic disease, current and previous treatment for atopic eczema and presence of *FLG* LOFM, patients who were vitamin D deficient had an increased risk of having moderate‐severe eczema, as classified by EASI score.

Our data showed after adjusting for confounding and for multiple testing, lowest 25(OH)D_3_ was associated with EASI score at recruitment but nearest 25(OH)D_3_ was not. As this is a retrospective study, our participants' 25(OH)D_3_ levels could be a biomarker for levels of UV exposure. In addition, low UV‐light exposure in people with non‐white skin phototypes may be related to higher EASI scores. Vitamin D supplementation for eczema is controversial, with several studies inconclusive. A recent systematic review and meta‐analysis demonstrated an overall positive effect of vitamin D supplementation on atopic dermatitis severity.^7^

There is a lack of research on eczema severity in non‐white populations, and most current studies only discuss white skin or do not mention ethnicity.^8^, ^20^ The majority (84.3%) of our participants had a history of deficient or insufficient 25(OH)D_3_, evidenced by lowest 25(OH)D_3_, supporting that vitamin D is low in people with pigmented skin living in low UV regions.^4^, ^6^ Vitamin D3 and its metabolites regulate skin barrier function through synthesis of proteins such as filaggrin, keratinocyte differentiation and regulation of antimicrobial peptides, such as cathelicidins.^21^ Oral vitamin D3 supplementation in children with atopic eczema reduces AD severity through increasing the epidermal expression of the vitamin D receptor and cathelicidin in lesional skin.^22^ NICE and RCPCH recommend treatment of vitamin D deficiency in children for 8–12 weeks (colecalciferol 3000IU daily aged 1–5 months, 6000IU daily aged 6months‐11 years, 10,000IU daily aged 12–17 years), while they recommend supplementation of vitamin D (colecalciferol 400–600IU daily) for children with vitamin D insufficiency.^23^

The presence of *FLG* LOFM has been linked to higher vitamin D levels, with the suggestion that the presence of these variants favours intracutaneous vitamin D3 production in people with Northern European ancestry.^24^, ^25^ We do not see this relationship in our cohort, despite an enrichment of *FLG* mutations with 37.5% (204/551) having at least one *FLG* LOFM. This may be due to our population being at high risk of vitamin D deficiency/insufficiency, or due to the presence of different *FLG* variants in Asian populations compared to white European populations.^10^

The EASI score has been validated for the assessment of atopic eczema extent and severity.^15^ Assessment of erythema in atopic dermatitis is more difficult in skin of colour, however the EASI score is recommended as the optimal core measure of eczema severity for patients with all skin colours.^26^ Our data supports that EASI score is associated with eczema severity. This is demonstrated by associations with higher EASI scores and use of potent or very potent topical corticosteroid on the body, or treatment of eczema on the face. Those with higher EASI scores were more likely to have had admissions with complications of eczema and were more likely to be on immunosuppression/biologic therapy, therefore they were more likely to have had blood testing, including 25(OH)D_3_.

This study has the advantage of assessing the relationship between EASI score and serum 25(OH)D_3_ in a large cohort of Bangladeshi patients, who are under‐studied in existing research into the role of vitamin D supplementation in eczema. Due to the nature of using a specific cohort we cannot be sure this would be generalisable to other populations living in the UK, or in lower latitude regions. Limitations include a lack of data on supplementation and dietary intake of vitamin D, and whether vitamin D supplementation resulted in a decrease in EASI score. Eczema severity including EASI score, Patient Orientated Eczema Measure and Dermatology Life Quality Index/Children's Dermatology Life Quality Index were assessed in a single visit and may not be truly representative of each patient's average eczema severity as most of the patients were on topical treatment.

## 5 CONCLUSION

Vitamin D deficiency is common in this cohort of Bangladeshi children and young adults living in East London, regardless of eczema severity. The findings demonstrate a weak, inverse correlation between serum 25(OH)D_3_ and eczema severity in the THEA cohort. Further research on the role of vitamin D supplementation in atopic eczema, particularly in people with non‐white skin phototypes living in low UV regions, should be performed.

## CONFLICT OF INTEREST STATEMENT

Rebecca L. McCarthy: Position funded by Palvella Therapeutics to work on a clinical trial. Unrelated to this work and all funding goes to the university. Edel A. O'Toole: Research funding: Kamari Pharma, Unilever. Consultancy: Azitra Inc, Palvella Therapeutics and Kamari Pharma; Speaker: Almirall. All unrelated to this work and all funding goes to the university.

## AUTHOR CONTRIBUTIONS

**Rebecca L. McCarthy:** Conceptualization (equal); Data curation (equal); Formal analysis (lead); Investigation (equal); Methodology (equal); Project administration (lead); Writing – original draft (lead); Writing – review & editing (lead). **Soha S. Tawfik:** Data curation (supporting); Formal analysis (supporting); Methodology (supporting); Visualization (supporting); Writing – original draft (supporting); Writing – review & editing (supporting). **Ioannis Theocharopoulos:** Conceptualization (equal); Formal analysis (supporting); Investigation (supporting); Methodology (supporting); Writing – original draft (supporting); Writing – review & editing (supporting). **Ravinder Atkar:** Conceptualization (equal); Data curation (equal); Investigation (equal); Supervision (supporting); Writing – review & editing (supporting). **Bryan McDonald:** Conceptualization (equal); Data curation (equal); Investigation (equal); Supervision (supporting); Writing – review & editing (supporting). **Sasha Dhoat:** Conceptualization (equal); Data curation (equal); Investigation (equal); Supervision (supporting); Writing – review & editing (supporting). **Aaron Hughes:** Conceptualization (supporting); Data curation (supporting); Formal analysis (supporting); Visualization (supporting); Writing – original draft (supporting); Writing – review & editing (supporting). **Bjorn R. Thomas:** Conceptualization (equal); Data curation (equal); Formal analysis (equal); Funding acquisition (equal); Investigation (equal); Methodology (equal); Project administration (equal); Supervision (supporting); Visualization (supporting); Writing – original draft (supporting); Writing – review & editing (supporting). **Edel A. O'Toole:** Conceptualization (lead); Data curation (lead); Funding acquisition (lead); Investigation (lead); Methodology (lead); Resources (lead); Supervision (lead); Writing – original draft (supporting); Writing – review & editing (supporting).

## ETHICS STATEMENT

Ethical approval was granted through the Health Research Authority after review by the Hampstead Regional Ethics Committee (REC 18/LO/0017; ReDA reference: 011978).

## Supporting information

Supplementary Material

## Data Availability

The data that support the findings of this study are available from the corresponding author upon reasonable request.
