# Supplementary material for: Vitamin D deficiency and atopic dermatitis severity in a Bangladeshi population living in East London: A cross‐sectional study
Source: Skin Health Dis. 2024 Mar 12;4(3):e358. doi: 10.1002/ski2.358 (PMC11150754; doi:10.1002/ski2.358)
Supplement: Supplementary file 1 — Supplementary Material [file SKI2-4-e358-s001.docx]

**Supporting information**

**Table S1:** Demographic features by EASI score dichotomized at 7

| **Characteristic** | **N** | **Overall**  N = 681*^1^* | **Eczema severity** | | ***p*-value***^2^* | **q-value***^3^* |
| --- | --- | --- | --- | --- | --- | --- |
|  |  |  | **≤7**  N = 421*^1^* | **>7**  N = 260*^1^* |  |  |
| **Lowest serum 25(OH)D3** | 338 | 21.0 (13.0, 37.8) | 22.5 (15.0, 43.8) | 17.0 (10.0, 31.2) | **<0.001** | **0.001** |
| Missing |  | 343 | 227 | 116 |  |  |
| **Nearest serum 25(OH)D3** | 338 | 31.0 (19.0, 51.0) | 33.0 (21.0, 55.8) | 29.0 (17.0, 47.2) | **0.044** | >0.99 |
| Missing |  | 343 | 227 | 116 |  |  |
| **Lowest 25(OH)D_3_ (nmol/L)** | 681 |  |  |  | **<0.001** | **0.017** |
| ≥ 50 (sufficient) |  | 204 (30%) | 104 (25%) | 100 (38%) |  |  |
| 25 – 50 (insufficient) |  | 81 (12%) | 50 (12%) | 31 (12%) |  |  |
| < 25 (deficient) |  | 53 (7.8%) | 40 (9.5%) | 13 (5.0%) |  |  |
| Not tested |  | 343 (50%) | 227 (54%) | 116 (45%) |  |  |
| **Nearest 25(OH)D_3_ (nmol/L)** | 681 |  |  |  | **0.032** | 0.81 |
| ≥ 50 (sufficient) |  | 130 (19%) | 69 (16%) | 61 (23%) |  |  |
| 25 – 50 (insufficient) |  | 117 (17%) | 66 (16%) | 51 (20%) |  |  |
| < 25 (deficient) |  | 91 (13%) | 59 (14%) | 32 (12%) |  |  |
| Not tested |  | 343 (50%) | 227 (54%) | 116 (45%) |  |  |
| **age** | 681 | 9.5 (4.8, 14.5) | 8.8 (4.6, 14.2) | 10.4 (5.8, 15.2) | **0.014** | 0.35 |
| **Male sex** | 681 | 391 (57%) | 230 (55%) | 161 (62%) | 0.062 | >0.99 |
| **BMI** | 673 | 17.9 (15.6, 21.9) | 17.7 (15.7, 21.7) | 18.1 (15.6, 22.5) | 0.38 | >0.99 |
| Missing |  | 8 | 5 | 3 |  |  |
| **Country of birth** | 681 |  |  |  | 0.054 | >0.99 |
| UK |  | 660 (97%) | 413 (98%) | 247 (95%) |  |  |
| Bangladesh |  | 3 (0.4%) | 1 (0.2%) | 2 (0.8%) |  |  |
| Other |  | 18 (2.6%) | 7 (1.7%) | 11 (4.2%) |  |  |
| **ESEC** | 679 |  |  |  | 0.36 | >0.99 |
| Intermediate |  | 189 (28%) | 110 (26%) | 79 (31%) |  |  |
| Salariat |  | 146 (22%) | 96 (23%) | 50 (19%) |  |  |
| Working |  | 344 (51%) | 214 (51%) | 130 (50%) |  |  |
| Missing |  | 2 | 1 | 1 |  |  |
| **EASI score** | 681 | 4.3 (1.5, 10.4) | 2.2 (0.9, 3.9) | 13.2 (9.3, 21.6) | **<0.001** | **<0.001** |
| **POEM score** | 681 | 13.0 (8.0, 19.0) | 10.0 (5.0, 16.0) | 18.0 (13.0, 22.0) | **<0.001** | **<0.001** |
| **DLQI** | 110 | 7.0 (3.0, 14.0) | 5.0 (2.0, 8.0) | 13.0 (6.0, 18.0) | **<0.001** | **<0.001** |
| Missing |  | 571 | 358 | 213 |  |  |
| **CDLQI** | 430 | 9.0 (5.0, 14.0) | 7.0 (4.0, 12.0) | 12.0 (7.0, 16.8) | **<0.001** | **<0.001** |
| Missing |  | 251 | 157 | 94 |  |  |
| **Admission to hospital with eczema flare** | 679 | 48 (7.1%) | 19 (4.5%) | 29 (11%) | **<0.001** | **0.025** |
| Missing |  | 2 | 1 | 1 |  |  |
| **Admission to hospital with eczema herpeticum** | 680 | 99 (15%) | 47 (11%) | 52 (20%) | **0.001** | **0.034** |
| Missing |  | 1 | 0 | 1 |  |  |
| **Admission to hospital with bacterial infection** | 680 | 46 (6.8%) | 15 (3.6%) | 31 (12%) | **<0.001** | **<0.001** |
| Missing |  | 1 | 0 | 1 |  |  |
| **Current immunosuppressive therapy** | 681 | 34 (5.0%) | 8 (1.9%) | 26 (10%) | **<0.001** | **<0.001** |
| **Previous immunosuppressive therapy** | 681 |  |  |  | **<0.001** | **<0.001** |
| Never |  | 605 (89%) | 393 (93%) | 212 (82%) |  |  |
| Previous or current |  | 76 (11%) | 28 (6.7%) | 48 (18%) |  |  |
| **Current prophylactic antibiotics or antivirals** | 681 | 20 (2.9%) | 6 (1.4%) | 14 (5.4%) | **0.003** | 0.074 |
| **Topical anti-inflammatory treatment on head/neck** | 681 |  |  |  | **<0.001** | **<0.001** |
| No anti-inflammatory treatment |  | 269 (40%) | 207 (49%) | 62 (24%) |  |  |
| Mild - moderate steroid |  | 305 (45%) | 155 (37%) | 150 (58%) |  |  |
| Potent - very potent steroid |  | 51 (7.5%) | 29 (6.9%) | 22 (8.5%) |  |  |
| Calcineurin inhibitor |  | 49 (7.2%) | 27 (6.4%) | 22 (8.5%) |  |  |
| Other |  | 7 (1.0%) | 3 (0.7%) | 4 (1.5%) |  |  |
| **Topical anti-inflammatory treatment on body** | 681 |  |  |  | **<0.001** | **<0.001** |
| No anti-inflammatory treatment |  | 179 (26%) | 141 (33%) | 38 (15%) |  |  |
| Mild - moderate steroid |  | 99 (15%) | 77 (18%) | 22 (8.5%) |  |  |
| Potent - very potent steroid |  | 390 (57%) | 196 (47%) | 194 (75%) |  |  |
| Calcineurin inhibitor |  | 6 (0.9%) | 4 (1.0%) | 2 (0.8%) |  |  |
| Other |  | 7 (1.0%) | 3 (0.7%) | 4 (1.5%) |  |  |
| **Physician diagnosed asthma** | 681 | 171 (25%) | 100 (24%) | 71 (27%) | 0.30 | >0.99 |
| **Physician diagnosed hayfever** | 681 | 214 (31%) | 131 (31%) | 83 (32%) | 0.83 | >0.99 |
| **Physician diagnosed food allergy** | 681 | 276 (41%) | 168 (40%) | 108 (42%) | 0.67 | >0.99 |
| **FLG LOFM** | 681 |  |  |  | 0.12 | >0.99 |
| None |  | 347 (51%) | 219 (52%) | 128 (49%) |  |  |
| At least one |  | 204 (30%) | 115 (27%) | 89 (34%) |  |  |
| Not tested |  | 130 (19%) | 87 (21%) | 43 (17%) |  |  |
| *^1^* Median (IQR); n (%) | | | | | | |
| *^2^* Wilcoxon rank sum test; Pearson's Chi-squared test; Fisher's exact test | | | | | | |
| *^3^* Bonferroni correction for multiple testing  ESEC, European Socioeconomic Class; EASI, Eczema Area and Severity Index; POEM, Patient Orientated Eczema Measure; FLG LOFM, FLG loss of function mutation | | | | | | |

**Table S2:** Demographic features by nearest recorded vitamin D3 level

| Characteristic | N | Overall  N = 681*^1^* | Nearest vitamin D level | | | | p-value*^2^* |
| --- | --- | --- | --- | --- | --- | --- | --- |
|  |  |  | **> 50**  N = 91*^1^* | **25 – 50**  N = 117*^1^* | **< 25**  N = 130*^1^* | **Not tested**  N = 343*^1^* |  |
| Age | 681 | 9.5  (4.8, 14.5) | 9.3  (5.3, 13.2) | 10.6  (7.7, 14.8) | 12.9  (9.1, 15.7) | 7.2  (3.0, 13.2) | **<0.001** |
| Male sex | 681 | 391 (57%) | 50 (55%) | 70 (60%) | 77 (59%) | 194 (57%) | 0.85 |
| BMI | 673 | 17.9  (15.6, 21.9) | 17.8  (15.5, 20.6) | 18.6  (16.1, 22.9) | 20.0  (16.1, 23.8) | 17.4  (15.3, 21.9) | **0.004** |
| Missing |  | 8 | 2 | 1 | 1 | 4 |  |
| ESEC | 679 |  |  |  |  |  | **0.009** |
| Working |  | 344 (51%) | 43 (47%) | 64 (55%) | 64 (50%) | 173 (50%) |  |
| Intermediate |  | 189 (28%) | 28 (31%) | 23 (20%) | 50 (39%) | 88 (26%) |  |
| Salariat |  | 146 (22%) | 20 (22%) | 29 (25%) | 15 (12%) | 82 (24%) |  |
| Missing |  | 2 | 0 | 1 | 1 | 0 |  |
| History of atopic disease | 681 | 509 (75%) | 75 (82%) | 94 (80%) | 114 (88%) | 226 (66%) | **<0.001** |
| EASI score | 681 | 4.3  (1.5, 10.4) | 4.0  (1.9, 9.6) | 5.2  (2.0, 12.2) | 6.8  (2.7, 14.9) | 3.7  (1.2, 9.0) | **<0.001** |
| EASI score category | 681 |  |  |  |  |  | **0.032** |
| ≤ 7 |  | 421 (62%) | 59 (65%) | 66 (56%) | 69 (53%) | 227 (66%) |  |
| >7 |  | 260 (38%) | 32 (35%) | 51 (44%) | 61 (47%) | 116 (34%) |  |
| EASI score category | 681 |  |  |  |  |  | **<0.001** |
| ≤ 10 |  | 503 (74%) | 69 (76%) | 81 (69%) | 80 (62%) | 273 (80%) |  |
| > 10 |  | 178 (26%) | 22 (24%) | 36 (31%) | 50 (38%) | 70 (20%) |  |
| Admission with eczema herpeticum | 680 | 99 (15%) | 15 (16%) | 23 (20%) | 25 (19%) | 36 (11%) | **0.024** |
| Missing |  | 1 | 0 | 0 | 0 | 1 |  |
| Admission with bacterial infection | 680 | 46 (6.8%) | 8 (8.8%) | 10 (8.5%) | 14 (11%) | 14 (4.1%) | **0.039** |
| Missing |  | 1 | 0 | 0 | 0 | 1 |  |
| Admission with eczema flare | 679 | 48 (7.1%) | 9 (9.9%) | 10 (8.5%) | 8 (6.2%) | 21 (6.2%) | 0.55 |
| Missing |  | 2 | 0 | 0 | 0 | 2 |  |
| FLG LOFM | 681 |  |  |  |  |  | 0.093 |
| None |  | 347 (51%) | 45 (49%) | 64 (55%) | 65 (50%) | 173 (50%) |  |
| At least one |  | 204 (30%) | 26 (29%) | 36 (31%) | 49 (38%) | 93 (27%) |  |
| Not tested |  | 130 (19%) | 20 (22%) | 17 (15%) | 16 (12%) | 77 (22%) |  |
| *^1^* Median (IQR); n (%)  *^2^* Kruskal-Wallis rank sum test; Pearson's Chi-squared test  ESEC, European Socioeconomic Class; EASI, Eczema Area and Severity Index; POEM, Patient Orientated Eczema Measure; FLG LOFM, FLG loss of function mutation | | | | | | | |

**Table S3:** EASI score and serum vitamin D levels by FLG loss of function mutation status

| **Characteristic** | **N** | **Overall**  N = 682*^1^* | ***FLG* LOFM** | | | ***p*-value***^2^* |
| --- | --- | --- | --- | --- | --- | --- |
|  |  |  | **None**  N = 347*^1^* | **At least one**  N = 204*^1^* | **Not tested**  N = 131*^1^* |  |
| **EASI** | 681 | 4.3 (1.5, 10.4) | 4.0 (1.4, 10.0) | 6.0 (2.6, 11.7) | 3.8 (1.2, 9.7) | **0.008** |
| **Lowest 25(OH)D3 nmol/L** | 339 | 21.0 (13.0, 38.0) | 19.5 (12.0, 33.5) | 22.0 (12.5, 37.0) | 28.5 (13.0, 44.8) | 0.15 |
| Missing |  | 343 | 173 | 93 | 77 |  |
| **Nearest 25(OH)D3 nmol/L** | 339 | 31.0 (19.0, 51.0) | 31.0 (20.0, 50.0) | 29.0 (18.5, 46.5) | 40.5 (20.2, 58.8) | 0.34 |
| Missing |  | 343 | 173 | 93 | 77 |  |
| *^1^* Median (IQR), *^2^* Kruskal-Wallis rank sum test  *FLG* LOFM*,* Filaggrin loss of function mutation; EASI, Eczema Area and Severity Index | | | | | | |

**Table S4:** Odds of EASI > 7 using lowest recorded vitamin D3 level versus:

| **Characteristic** | **OR** | **95% CI** | **p-value** |
| --- | --- | --- | --- |
| **Lowest 25(OH)D_3_ (nmol/L)** |  |  |  |
| ≥ 50 (sufficient) | Ref | | |
| 25 – 50 (insufficient) | 1.53 | 0.67, 3.60 | 0.3 |
| < 25 (deficient) | 2.07 | 0.99, 4.53 | 0.060 |
| Not tested | 1.64 | 0.82, 3.45 | 0.2 |
| **Age** | 1.02 | 0.98, 1.06 | 0.3 |
| **Male sex** | 1.32 | 0.93, 1.87 | 0.12 |
| **BMI** | 1.00 | 0.95, 1.04 | 0.8 |
| **History of atopic disease** | 0.69 | 0.45, 1.06 | 0.088 |
| **Current immunosuppressive therapy** | 2.58 | 1.08, 6.73 | **0.040** |
| **Previous immunosuppressive therapy** | 1.71 | 0.94, 3.12 | 0.078 |
| **Topical anti-inflammatory treatment on head/neck** |  |  |  |
| No anti-inflammatory treatment | Ref | | |
| Mild - moderate steroid | 2.63 | 1.69, 4.11 | **<0.001** |
| Potent - very potent steroid | 1.91 | 0.96, 3.79 | 0.064 |
| Calcineurin inhibitor | 1.96 | 0.95, 4.01 | 0.065 |
| Other | 4.25 | 0.75, 25.0 | 0.093 |
| **Topical anti-inflammatory treatment on body** |  |  |  |
| No anti-inflammatory treatment | Ref | | |
| Mild - moderate steroid | 0.77 | 0.39, 1.48 | 0.4 |
| Potent - very potent steroid | 2.17 | 1.33, 3.57 | **0.002** |
| Calcineurin inhibitor | 0.74 | 0.09, 4.43 | 0.7 |
| Other | 2.85 | 0.56, 15.9 | 0.2 |
| **ESEC** |  |  |  |
| Working | Ref | | |
| Intermediate | 1.18 | 0.79, 1.77 | 0.4 |
| Salariat | 1.05 | 0.67, 1.65 | 0.8 |
| **Any *FLG* LOFM** |  |  |  |
| None | Ref | | |
| At least one | 1.37 | 0.93, 2.03 | 0.12 |
| Not tested | 0.87 | 0.53, 1.40 | 0.6 |
| **No. Obs.** | 671 |  |  |
| **Log-likelihood** | -390 |  |  |
| **AIC** | 824 |  |  |
| **BIC** | 924 |  |  |
| *FLG* LOFM*,* Filaggrin loss of function mutation; OR, Odds ratio; 95% CI, 95% confidence interval; AIC, Akaike Information Criterion; BIC, Bayesian Information Criterion; EASI, Eczema Area and Severity Index; Ref, reference level for categorical variables in logistic regression | | | |

**Table S5:** Odds ratio of EASI > 10 and EASI > 7 using nearest recorded vitamin D3 level versus:

|  | EASI > 10 | | | EASI > 7 | | |
| --- | --- | --- | --- | --- | --- | --- |
| Characteristic | **OR** | **95% CI** | ***p*-value** | **OR** | **95% CI** | ***p*-value** |
| Nearest vitamin D3 (nmol/L) |  |  |  |  |  |  |
| ≥ 50 (sufficient) | Ref | | | Ref | | |
| 25 – 50 (insufficient) | 1.54 | 0.77, 3.15 | 0.2 | 1.40 | 0.74, 2.66 | 0.3 |
| < 25 (deficient) | 2.05 | 1.03, 4.17 | **0.043** | 1.49 | 0.79, 2.84 | 0.2 |
| Not tested | 1.25 | 0.67, 2.41 | 0.5 | 1.27 | 0.73, 2.25 | 0.4 |
| Age | 1.04 | 1.00, 1.09 | 0.054 | 1.03 | 0.99, 1.07 | 0.2 |
| Male sex | 1.41 | 0.96, 2.10 | 0.086 | 1.31 | 0.92, 1.86 | 0.13 |
| BMI | 0.98 | 0.93, 1.02 | 0.4 | 0.99 | 0.95, 1.04 | 0.8 |
| History of atopic disease | 0.87 | 0.54, 1.41 | 0.6 | 0.70 | 0.46, 1.06 | 0.10 |
| Current immunosuppressive therapy | 2.03 | 0.89, 4.72 | 0.094 | 2.97 | 1.25, 7.74 | **0.018** |
| Previous immunosuppressive therapy | 1.87 | 1.02, 3.42 | **0.041** | 1.73 | 0.96, 3.16 | 0.069 |
| Topical anti-inflammatory treatment on head/neck |  |  |  |  |  |  |
| None | Ref | | | Ref | | |
| Mild - moderate steroid | 3.10 | 1.86, 5.30 | **<0.001** | 2.63 | 1.70, 4.12 | **<0.001** |
| Potent - very potent steroid | 2.26 | 1.03, 4.85 | **0.037** | 1.95 | 0.98, 3.87 | 0.056 |
| Calcineurin inhibitor | 2.97 | 1.35, 6.45 | **0.006** | 2.01 | 0.98, 4.11 | 0.055 |
| Other | 2.14 | 0.21, 13.5 | 0.5 | 4.37 | 0.76, 25.7 | 0.088 |
| Topical anti-inflammatory treatment on body |  |  |  |  |  |  |
| None | Ref | | | Ref | | |
| Mild - moderate steroid | 0.71 | 0.30, 1.62 | 0.4 | 0.79 | 0.40, 1.52 | 0.5 |
| Potent - very potent steroid | 2.10 | 1.17, 3.85 | **0.014** | 2.18 | 1.34, 3.58 | **0.002** |
| Calcineurin inhibitor | 1.27 | 0.16, 7.83 | 0.8 | 0.81 | 0.10, 4.82 | 0.8 |
| Other | 1.65 | 0.21, 9.46 | 0.6 | 2.84 | 0.56, 16.0 | 0.2 |
| ESEC |  |  |  |  |  |  |
| Working | Ref | | | Ref | | |
| Intermediate | 1.38 | 0.89, 2.14 | 0.15 | 1.17 | 0.78, 1.75 | 0.4 |
| Salariat | 0.90 | 0.53, 1.50 | 0.7 | 1.03 | 0.66, 1.62 | 0.9 |
| *FLG* LOFM |  |  |  |  |  |  |
| None | Ref | | | Ref | | |
| At least one | 1.27 | 0.83, 1.96 | 0.3 | 1.36 | 0.92, 2.01 | 0.13 |
| Not tested | 1.04 | 0.60, 1.76 | 0.9 | 0.86 | 0.53, 1.38 | 0.5 |
| No. Obs. | 671 |  |  | 671 |  |  |
| Log-likelihood | -330 |  |  | -391 |  |  |
| AIC | 703 |  |  | 827 |  |  |
| BIC | 802 |  |  | 926 |  |  |
| *FLG* LOFM*,* Filaggrin loss of function mutation; OR, Odds ratio; 95% CI, 95% confidence interval; AIC, Akaike Information Criterion; BIC, Bayesian Information Criterion; EASI, Eczema Area and Severity Index; Ref, reference level for categorical variables in logistic regression | | | | | | |


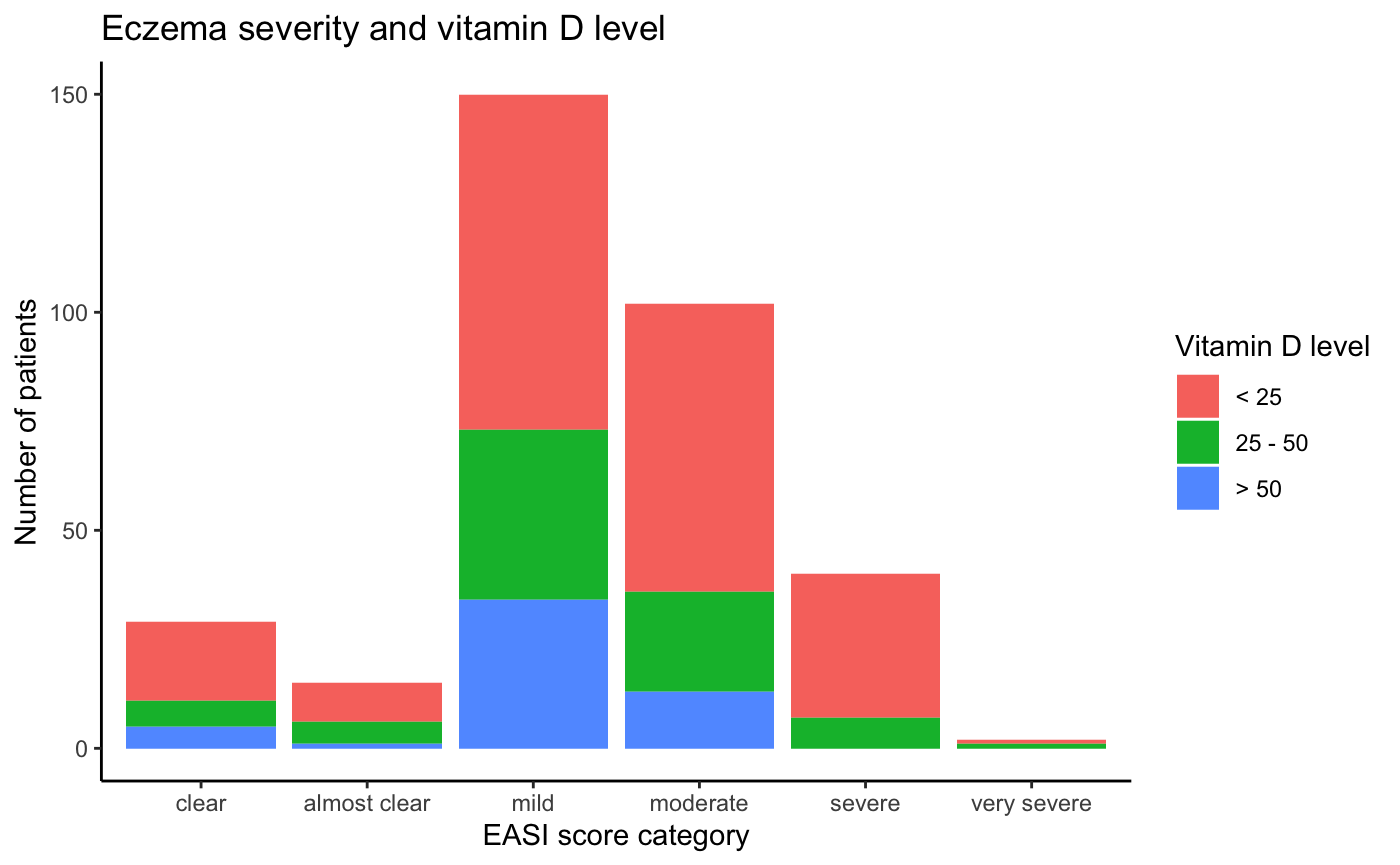


**Supplementary figure 1:** Distribution of eczema severity by EASI (eczema area and severity index) score category and serum vitamin D levels (deficient (<25nmol/L), insufficient (25-50nmol/L) and sufficient (>50 nmol/L).)


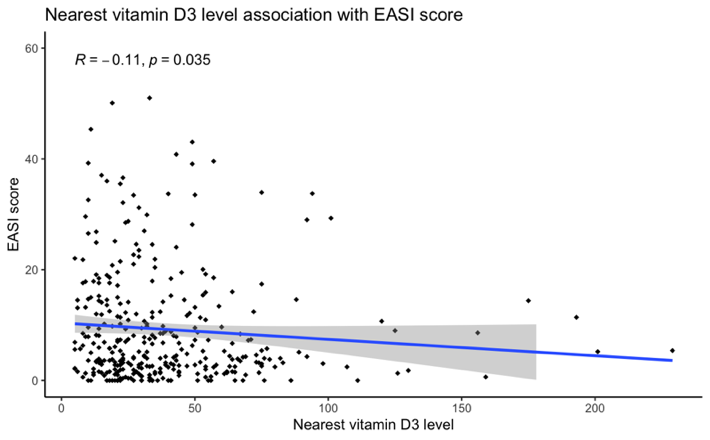


**Supplementary figure 2**: Nearest recorded 25(OH)D_3_ at time of recruitment is inversely correlated with EASI score (Spearman’s rank R^2^ = -0.22, *p* < 0.001).


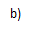


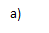


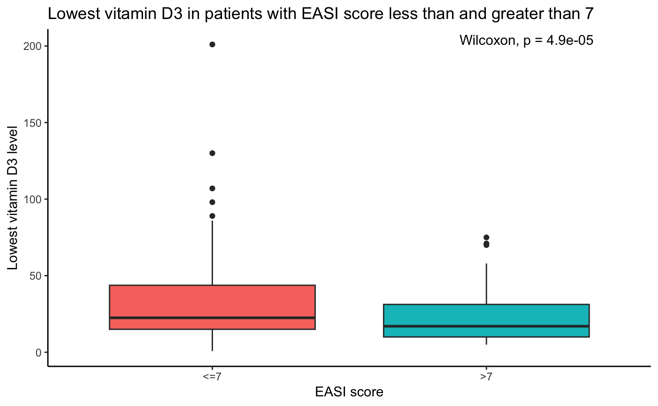

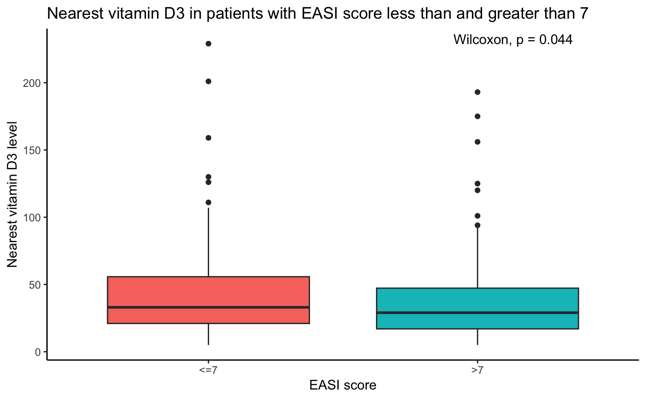


**Supplementary figure 3:** Box plots of (a) lowest and (b) nearest vitamin D3 levels in participants with EASI ≤ 7 and EASI > 7.

**Appendix S1: Additional Materials and Methods**

*Saliva collection for genomic DNA*

A sample of saliva was used to collect DNA for the project. (GeneFixTM DNA Saliva Collectors from Isohelix, Harrietsham, United Kingdom). Samples were collected and stored as per manufacturers recommendations. DNA extraction and targeted FLG sequencing was performed as detailed by Thomas *et al*.^14^

*Missing data*

Participants who had never had vitamin D3 testing (343/681) were included in analyses as a ‘not tested’ group, as they were not missing at random. There was missing sequencing data for 130/681 participants. Sixty-eight samples had poor sequencing coverage (<50) and sixty-two samples had no genetic testing performed or available. For the purposes of analyses, these samples were included in a ‘not tested’ group. Ten participants were excluded from logistic regression analysis as they did not have results available for other variables (BMI n=9, ESEC n=2).

*Statistical analysis*

Generalised linear models (GLMs) were used to investigate the associations between eczema severity (EASI), and the lowest and nearest recorded vitamin D3 levels. All analyses were performed using R (v. 4.2.2.) software. For logistic regression, the glm() function was used, with a binomial family. Results were exponentiated to create odds ratios for each variable in the model.

Adjusted models used age, sex, body mass index (BMI), socioeconomic class and plausible predictive variables: current topical anti-inflammatory treatment on the head/neck, current topical anti-inflammatory treatment on the body, current immunosuppressive medication, previous immunosuppressive medication, history of atopic disease and *FLG* LOFM status. BMI was included as increased BMI is associated with *FLG* variation. Social class was included to attempt to adjust for unknown factors related to social inequity. Social class was calculated using the European Socio-Economic Classification (ESeC). The ESeC score was collapsed into three groups: working class (ESeC 6-9), middle class (ESeC 4-6), higher class (ESeC 1-3) as per Rose and Harrison. Current topical and current/previous immunosuppressive therapy was included to adjust for treatment which would effect EASI score. History of atopic disease was included as it is associated with higher risk for developing eczema and asthma is linked to worse eczema severity. *FLG* LOFM status was included as *FLG* mutations are associated with worse eczema severity.
